# Polycystic ovary syndrome is a risk factor for sarcopenic obesity: a case control study

**DOI:** 10.1186/s12902-019-0381-4

**Published:** 2019-07-01

**Authors:** Laura E. McBreairty, Philip D. Chilibeck, Julianne J. Gordon, Donna R. Chizen, Gordon A. Zello

**Affiliations:** 1 College of Pharmacy and Nutrition, University of Saskatchewan, 104 Clinic Place, Saskatoon, SK S7N 2Z4 Canada; 2 College of Kinesiology, Physical Activity Complex, University of Saskatchewan, 87 Campus Drive, Saskatoon, SK S7N 5B2 Canada; 3 Obstetrics and Gynecology, College of Medicine, University of Saskatchewan, 103 Hospital Drive, Saskatoon, SK S7N 0W8 Canada

**Keywords:** Muscle, Insulin resistance, Vitamin D, Inflammation

## Abstract

**Background:**

Polycystic ovary syndrome (PCOS) is the most common endocrine disorder in young women and increases risk of cardiovascular and metabolic disease, and infertility. Women with PCOS share many characteristics commonly associated with aging including chronic inflammation and insulin resistance, which may be associated with “sarcopenic obesity”, a term used to describe low appendicular skeletal muscle mass relative to total body mass. The purpose of this work was to determine the prevalence of sarcopenic obesity in women with PCOS. We hypothesized there would be a high prevalence of sarcopenic obesity, and that % appendicular skeletal muscle mass and markers of inflammation and insulin resistance would be inversely correlated in this population.

**Methods:**

Dual energy X-ray absorptiometry was used to assess body composition in 68 women with PCOS aged 18-35y and 60 healthy age-matched women from the same geographic area. Sarcopenic obesity was defined as having % appendicular skeletal muscle mass 2 standard deviations below the mean for the healthy age-matched controls and a % body fat above 35%. Data were analyzed with Mann-Whitney U-tests and Spearman correlations.

**Results:**

53% of women with PCOS were classified as sarcopenic obese. Women with PCOS had a median (interquartile range) appendicular skeletal muscle mass of 23.8 (22.3–25.8)% which was lower than the control median of 30.4 (28.6–32.4)% (*p* < 0.0001). Among women with PCOS, there were negative correlations between % appendicular skeletal muscle mass and the homeostasis model assessment insulin resistance index (*r* = − 0.409; *p* < 0.01), high sensitivity C-reactive protein (*r* = − 0.608; *p* < 0.0001) and glycosylated hemoglobin (*r* = − 0.430; *p* < 0.0001). Furthermore, % appendicular skeletal muscle mass correlated positively with vitamin D (*r* = 0.398; *p* < 0.0001) in women with PCOS, which is thought to positively affect skeletal muscle mass.

**Conclusions:**

Women with PCOS have a high prevalence of sarcopenic obesity, which is correlated to insulin resistance and inflammation.

## Background

Polycystic Ovary Syndrome (PCOS) is a very common endocrine disorder in young women with a prevalence estimated to be 5–12% of premenopausal women [1]. Clinical features associated with the syndrome include menstrual and ovulatory dysfunction, hyperandrogenemia, hirsutism, polycystic ovaries, insulin resistance, and hyperinsulinism [2]. Women with PCOS are at an increased risk of developing infertility, type 2 diabetes mellitus, obesity and hypertension [1–4]. Approximately 30% of women with PCOS are obese [5], yet a modest weight loss of > 5% has been shown to improve insulin sensitivity and reproductive function, supporting the notion that obesity exacerbates characteristics of PCOS [6].

The term sarcopenic obesity, primarily used to describe many older individuals, is defined as low appendicular skeletal muscle mass (ASM) relative to total body mass [7]. Although the pathogenesis is not fully understood, sarcopenic obesity is thought to be associated with increased visceral adiposity. Adipose tissue releases inflammatory cytokines, which are associated with protein degradation and sarcopenia [8, 9]. Insulin resistance is also thought to be a contributing factor to sarcopenic obesity and may exacerbate skeletal muscle loss [10]. Although sarcopenic obesity is most commonly associated with the aging population, women with PCOS share many of the characteristics associated with aging. Women with PCOS have increased visceral adiposity compared to BMI-matched controls [11, 12]. Indicators of impaired glucose tolerance and insulin resistance such as homeostatic model assessment are also higher in women with PCOS [13, 14]. Chronic inflammation is also prevalent in this population with higher levels of inflammatory cytokines compared to controls [15, 16]. Despite a higher prevalence of these risk factors for sarcopenic obesity the prevalence of sarcopenic obesity in women with PCOS is currently unknown.

The primary objective of this study was to determine the prevalence of sarcopenic obesity in a sample of women with PCOS. Furthermore, we wanted to determine whether the metabolic measures associated with sarcopenic obesity in older adults were also associated with sarcopenic obesity in women with PCOS. We hypothesized that women with PCOS would have a high prevalence of sarcopenic obesity and that ASM would correlate negatively with markers of inflammation and insulin resistance (i.e. high sensitivity C-reactive protein [hsCRP], and the homeostasis model assessment insulin resistance index [HOMA]).

## Methods

The results in the present report are baseline measures from 68 participants involved in a larger study that determined the effect of a pulse-based diet (e.g., beans, lentils, chick-peas) and exercise intervention in women with PCOS [17]. Sixty healthy age-matched control women from the same city (i.e. Saskatoon SK, Canada) were also recruited to serve as a reference group to determine the prevalence of sarcopenic obesity in women with PCOS. All participants were between 18 and 35 years of age. Control participants were recruited via postings on the University of Saskatchewan website requesting participation from healthy females between 18 and 35 years old with regular menstrual cycles. Controls were excluded if they had any self-identified chronic conditions or irregular menstrual cycles. Women with PCOS were recruited via posters, contacting doctors’ offices and postings on the university website. To be included in the control group, women must not have been diagnosed with any chronic medical conditions, must not have been pregnant and must have had regular menstrual cycles. In the PCOS group, a diagnosis of PCOS was made by an obstetrician-gynecologist using criteria specified in the PCOS report of the Androgen Excess-PCOS Society Task Force [2]. A diagnosis of PCOS required 1) either oligo-amenorrhea and/or polycystic ovaries defined as > 25 follicles visualized by transvaginal ultrasonography to reflect the newest guidelines for polycystic ovaries (PCO) recommended by the Androgen Excess and Polycystic Ovary Syndrome Society, and 2) hyperandrogenism as defined by a Ferriman and Gallwey score of > 6 and/or biochemical hyperandrogenemia [2, 18, 19]. Women with PCOS associated with the following conditions were excluded from the study: Taking anti-seizure or anti-psychotic medications known to induce development of PCO; untreated hyperprolactinemia or thyroid disease; or, excessive adrenal androgen production confirmed by a diagnosis of congenital adrenal hyperplasia, Cushing’s disease or syndrome, or an adrenal tumor. Exclusion criteria for the PCOS study group included the use of hormonal birth control methods or fertility medications during the prior 3 months, any uncontrolled medical condition that interfered with ovarian or systemic hormone production, or women who were pregnant or breastfeeding. The screening for the PCOS study group was part of a diet and exercise intervention so exclusion criteria also included residing outside of the local geographic area or women with a medical condition limiting exercise or consumption of a pulse-based diet (allergies or intolerances). The study was approved by the University of Saskatchewan Biomedical Research Ethics Board and all women gave written informed consent before participation in the study.

Body composition was assessed using dual-energy X-ray absorptiometry (Hologic© Discovery Wi; Bedford, MA), performed and analyzed by a certified nuclear medicine technologist. A quality-control phantom scan was performed daily. Coefficients of variation for fat and bone mineral-free lean tissue mass were 3 and 0.5% respectively. Body mass was determined with a calibrated scale and height determined with a standiometer. Body mass index (BMI) was determined as mass (kg) divided by height squared (m^2^). Waist circumference was measured after removing clothing from the abdomen, by locating the top of the iliac crest and measuring horizontally at the tip of the landmark.

Percent ASM was calculated as the total bone mineral-free lean tissue mass of the arms and legs divided by total body mass times 100. Sarcopenic obesity was defined as having a %ASM two standard deviations (SD) below the mean value for the young sex-matched control group [20, 21] as well as a percentage body fat above 35% [22].

Blood was collected for the PCOS group following a 10 to 12 h fast and was centrifuged after a complete clot was formed. All samples were analyzed by the Saskatoon Health Region Laboratory in Saskatoon, Saskatchewan. Glucose and insulin levels were analyzed using the IMMULITE 2000 Systems Analyzer’s solid-phase enzyme-labelled chemiluminescent immunometric assay (Siemens, California). HOMA was calculated as fasting plasma insulin (pmol/L) * fasting plasma glucose (mmol/L)/22.5 * 10 (correction factor for SI units). The remaining metabolites were analyzed using Roche Cobas analyzers and kits (Cobas Roche Diagnostic, Basel, Switzerland) with glycosylated hemoglobin (HbA1C) analyzed using turbidimetric inhibition immunoassay, hsCRP analyzed using a particle enhanced immunoturbidimetric assay, and 25-hydroxyvitamin D analyzed using an electrochemiluminescence immunoassay. The intra-assay % coefficient of variation was < 5% for all assays. The limit of detection was 0.18 mmol/L for HbA1C, 0.15 mg/L for hsCRP, 7.5 nmol/L for vitamin D, and 0.11 mmol/L for glucose. Functional sensitivity was 0.3 mg/L for hsCRP and 4.01 ng/ml for Vitamin D.

The minimal sample size required was determined using the mean and SD of our control group for %ASM (30.2 ± 3.0%) and a predicted mean for sarcopenic obesity that was 2 SD lower (i.e. 24.1%, as a clinical cut-off for sarcopenic obesity [20–22]), a power of 90% and alpha of 0.05. This resulted in a required sample size of *n* = 7 per group.

Differences in characteristics between women with PCOS and control women were determined using a Mann-Whitney U-test as data did not follow a normal distribution. Women with PCOS were classified as sarcopenic obese according to the criteria of having a %ASM 2 SD below the mean for our female control group (*n* = 60), as well a % body fat above 35% [19–21]. All other women were classified as non-sarcopenic obese. Differences between sarcopenic obese and non-sarcopenic obese women with PCOS were determined using a Mann-Whitney U-test as data did not follow a normal distribution. Spearman correlations for anthropometric measures with metabolic markers were determined for all women with PCOS. Spearman correlations were also determined for %ASM and metabolic markers in both non-sarcopenic obese and sarcopenic obese women with PCOS, separately. All data were analyzed using Statistica version 12 (Statsoft, Chicago IL). Group comparisons are presented as median and interquartile range, with descriptive statistics of clinical blood measures and %ASM presented as means ± SD. A *P*-value < 0.05 was considered significant.

## Results

**Fig. 1:**
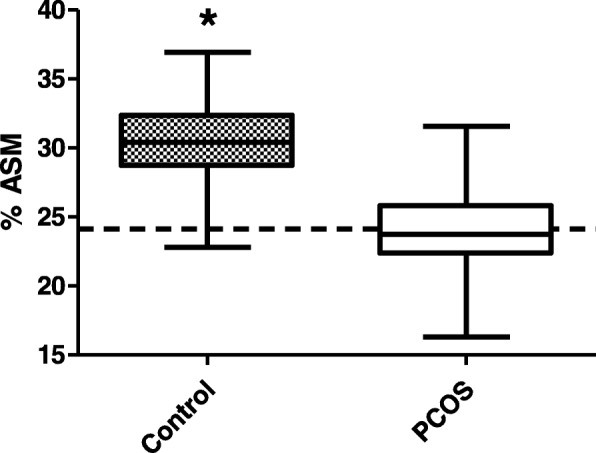
Percentage appendicular skeletal muscle mass (%ASM) in a young adult healthy female control population and women with polycystic ovary syndrome (PCOS). Data presented using box-and-whisker plot with central line representing median, box representing interquartile range and bars representing highest and lowest value. - - - represents 2 SD below mean % ASM in control population. (*) represents significant difference between groups, *p* < 0.0001)

**Fig. 2:**
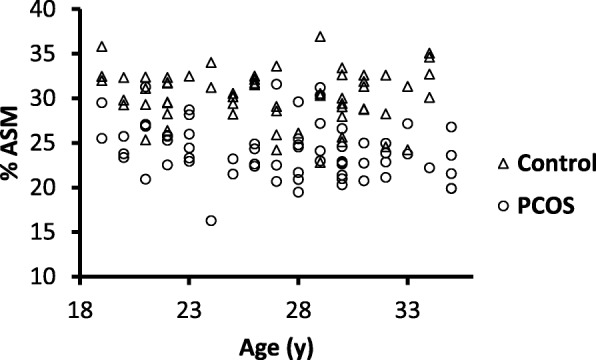
Percentage appendicular skeletal muscle mass (%ASM) versus age in women with polycystic ovary syndrome (PCOS) and healthy controls

The median BMI and age for the control group were 21.9 (20.5–23.8) kg/m^2^ and 27 (22.0–30.0) years, respectively. The median %ASM for the control group was 30.4 (28.6–32.4) % (Fig. 1). The mean %ASM for the control group was 30.2 ± 3.0% and the cut-off for sarcopenic obesity (defined as 2 SD below the mean of the control group) was 24.1% (Fig. 1). In women with PCOS the median BMI and age were 31.7 (25.6–37.4) kg/m^2^ and 28 (23.0–30.8) years, respectively. In women with PCOS the median %ASM was 23.8 (22.3–25.8) %; 53% of women with PCOS were defined as sarcopenic obese. Compared to controls, women with PCOS had a higher BMI (*p* < 0.0001) and lower %ASM (*p* < 0.0001) (Fig. 1), with no difference in age between the groups. Figure 2 shows %ASM plotted against age for women with PCOS and controls. The plot shows that across age groups, women with PCOS had consistently lower %ASM compared to controls.

The levels of Vitamin D, hsCRP, and HbA1C in women with PCOS were 61.9 ± 31.6 nmol • L^− 1^, 4.65 ± 4.77 mg • L^− 1^ and 5.32 ± 0.44%, respectively. HOMA in women with PCOS was 3.85 ± 3.88, which is categorized as insulin resistant [23]. Levels of hsCRP and HbA1C were in the normal clinical range in women with PCOS while levels of vitamin D were insufficient according to our hospital lab (Saskatoon Health region) where normal reference ranges of hsCRP and HbA1C are 0–7 mg • L^− 1^ and 4.5–6.5%, respectively, and normal ranges for vitamin D are 25–70 and 70–250 nmol • L^− 1^ for relative insufficient and optimal, respectively.

Percent ASM and percent bone-free lean tissue mass (% BFLTM) correlated negatively with hsCRP, HOMA, HbA1C, and positively with Vitamin D in women with PCOS (all *p* < 0.01) (Table 1). Percent total-body fat mass (% FM), BMI, and waist circumference correlated positively with hsCRP, HOMA and HbA1C, and negatively with Vitamin D in women with PCOS (all *p* < 0.01) (Table 1).

**Table 1:** Correlations between anthropometric measures and metabolic markers in women with polycystic ovary syndrome

	%ASM	%BFLTM	%FM	BMI	WC
hsCRP	−0.608**	−0.755**	0.733**	0.571**	0.628**
HOMA	−0.409*	−0.502**	0.520**	0.575**	0.581**
Vitamin D (25-OH)	0.398*	0.430**	−0.430**	−0.374*	−0.379*
HbA1C	−0.430**	−0.538**	0.532**	0.479**	0.410*

Correlation coefficient and *p* values determined using Spearman correlations. (*) represents significant correlation, *p* < 0.01, (**) represents significant correlation, *p* < 0.0001. % ASM, % appendicular skeletal muscle mass; % BFLTM, % bone-free lean tissue mass; % FM, % fat mass; BMI, body mass index; HbA1C, glycosylated hemoglobin; HOMA, homeostasis model assessment insulin resistance index hsCRP, high sensitive C-reactive protein; WC, waist circumference

**Fig. 3:**
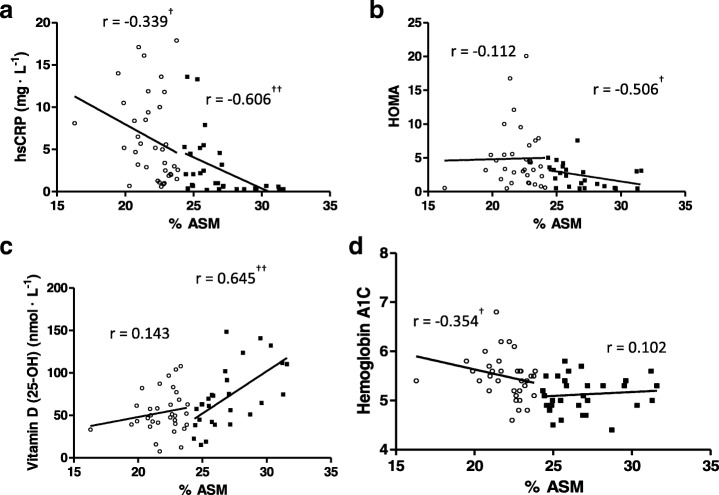
Correlations between % appendicular skeletal muscle mass (%ASM) and metabolic markers in sarcopenic obese (○) and non-sarcopenic obese (■) women with PCOS. (**a**) hsCRP, (**b**) HOMA, (**c**) Vitamin D, and (**d**) Hemoglobin A1C. Spearman correlations performed and presented for each group. (†) represents significant correlation (*p* < 0.05), (††) represents significant correlation (*p* < 0.001). hsCRP, high sensitive C-reactive protein; HOMA, homeostasis model assessment insulin resistance index

Among women with PCOS, 36 were classified as sarcopenic obese and 32 were non-sarcopenic obese. Sarcopenic obese women had a higher BMI and waist circumference as well as lower %ASM than non-sarcopenic obese women (*p* < 0.0001) (Table 2). Among women with PCOS, women with sarcopenic obesity also had higher levels of hsCRP (*p* < 0.0001), HOMA (*p* < 0.01), HbA1C (*p* < 0.01) and lower levels of Vitamin D (*p* < 0.01) (Table 2). In non-sarcopenic obese women with PCOS, %ASM remained negatively correlated with HOMA (*p* < 0.05) and hsCRP (*p* < 0.001), and positively correlated with Vitamin D (*p* < 0.001) (Fig. 3). In sarcopenic obese women with PCOS, %ASM remained negatively correlated with hsCRP (*p* < 0.05) and HbA1C (*p* < 0.05) (Fig. 3).

**Table 2:** Characteristics and metabolic markers in sarcopenic obese and non-sarcopenic obese women with polycystic ovary syndrome

	Non-Sarcopenic Obese	Sarcopenic Obese	Normal Clinical Range
BMI	27.1 (22.3–30.9)**	36.0 (30.4–39.7)	
%ASM	26.3 (25.0–28.3)**	22.5 (21.0–23.0)	
Waist Circumference (cm)	88.8 (78.8–100.4)**	108.9 (95.6–119.6)	
hsCRP (mg • L^−1^)	0.9 (0.4–4.5)**	5.1 (2.0–9.9)	0-7^a^
HOMA	2.38 (0.67–3.44)*	3.55 (1.74–5.90)	< 2.73^b^
Vitamin D (25-OH) (nmol • L^−1^)	63.8 (42.9–99.5)*	49.6 (39.1–65.6)	25–70 (relative insufficient); 70–250 (optimal)^a^
HbA1C (%)	5.1 (4.9–5.4)*	5.5 (5.2–5.6)	4.5–6.5^a^

Data are presented as median (interquartile range). (*) represents significant difference between groups, *p* < 0.01; (**) represents significant difference between groups, *p* < 0.0001. %ASM, % appendicular skeletal muscle mass; BMI, body mass index; HbA1C, glycosylated hemoglobin; HOMA, homeostasis model assessment insulin resistance index hsCRP, high sensitive C-reactive protein

^a^Saskatoon Health Region, Saskatoon, SK

^b^Cut off value for insulin resistance [20]

## Discussion

This study determined the prevalence of sarcopenic obesity to be 53% in our sample of women with PCOS. Among this population %ASM was positively associated with vitamin D and negatively associated with HOMA, hsCRP and HbA1C, with only hsCRP and HbA1C maintaining significance in sarcopenic obese women with PCOS. The prevalence of sarcopenic obesity was based on the percentage of women who had a %ASM lower than 24.1%, which was 2 SD below the mean for a young age-matched healthy female control group. This reference value is in keeping with other healthy young adult control groups from previous studies, which ranged from 21.1–25.1% [24, 25].

The term sarcopenic obesity as a classification [7] is not clearly defined in the literature and currently more than eight definitions can be found, leading to a highly variable prevalence range of 3.6–94% in older women [26]. Using similar criteria of %ASM and %body fat, the prevalence of sarcopenic obesity in a primarily Korean population is 3.3 and 12.5% in women aged 40–59 and ≥ 60, respectively [20], which is similar to the prevalence of 10.9% found in a Canadian population of older adults aged 68–82 years [22]. Although the pathogenesis of sarcopenic obesity is not fully understood, physical inactivity, increased visceral adiposity, increased inflammation, hormonal changes and insulin resistance are thought to be the primary factors associated with sarcopenic obesity [27]. From our results, women with PCOS may have a higher prevalence of sacropenic obesity than an older population; this is most likely due to the altered metabolic profile and increased inflammation associated with PCOS.

Both normal-weight and obese women with PCOS have increased visceral adiposity [11, 12], and visceral adiposity is associated with higher levels of inflammatory cytokines [8, 28], as well as the inflammatory marker hsCRP [29]. This body composition profile may in part explain the low grade chronic inflammation [30] and higher levels of hsCRP [15] associated with PCOS. In this study we used waist circumference as an indicator of visceral adiposity, which was positively associated with hsCRP, HOMA and HbA1C in women with PCOS. This is in keeping with another study which found a positive association between hsCRP and waist circumference in women with PCOS [31]. We also found a negative correlation between hsCRP and %ASM in women with PCOS which is consistent with studies in older adults [27] and obese individuals [32]. Inflammatory cytokines are associated with muscle catabolism and sarcopenia [9]. Furthermore, low grade inflammation negatively affects protein synthesis [33]. Thus, chronic inflammation in women with PCOS may be a contributing factor to the high prevalence of sarcopenic obesity in this population. Interestingly, although sarcopenic obesity has primarily been studied in older adults, it has been demonstrated that hsCRP has a stronger association with sarcopenic obesity in females less than 60 years old [34].

Insulin resistance is more prevalent in women with PCOS compared to both lean and overweight BMI-matched control groups [35], which may contribute to sarcopenic obesity in this population. In this study, HOMA was used as an indicator of insulin resistance and we demonstrated a negative correlation between HOMA scores and %ASM in women with PCOS. In addition, HbA1C, a marker of glycemic control, was negatively associated with %ASM in women with PCOS. Insulin is an anabolic hormone and insulin resistance is associated with accelerated muscle protein degradation [36]. Our results are supported by studies demonstrating a positive correlation between fat to lean mass ratio and HOMA in women with PCOS [37] as well as an association between percent lean mass with better insulin sensitivity in obese women [32]. Collectively, these studies and our data support the hypothesis that insulin resistance may contribute to sarcopenia in women with PCOS.

Vitamin D has recently received attention for the role it may play in positively affecting skeletal muscle growth and treatment of sarcopenia [38]. Although the existence of the vitamin D receptor (VDR) in muscle cells is still debated, it has been hypothesized that VDR signaling is important for normal muscle growth and vitamin D may have an anabolic effect by modulating intracellular signaling pathways and contributing to myoblast proliferation [38]. In this study, plasma 25-hydroxyvitamin D correlated positively with %ASM and negatively with waist circumference in women with PCOS. These results are consistent with others who have demonstrated a positive association between vitamin D and percent lean mass in obese individuals [32] and a negative association between vitamin D and waist circumference in women with PCOS [31]. Furthermore, vitamin D remains positively associated with skeletal muscle mass after adjusting for body fat mass in obese individuals [39]. A large proportion of the women with PCOS had low vitamin D levels (Table 2); however, this is typical of young Canadian women because of the limited sun exposure due to northern latitude. The proportion of women with PCOS with vitamin D level lower than 50 nmol/L in our study (41%) is identical to that of Canadians in the same age group from the general population [40].

In this study women with PCOS and sarcopenic obesity had a higher waist circumference and HOMA, as well as higher levels of hsCRP and HbA1C compared to women with PCOS but without sarcopenic obesity. Low grade chronic inflammation has been proposed as a primary contributor to sarcopenic obesity in older adults, particularly in women [41]. Interestingly, we demonstrated that in sarcopenic obese women with PCOS, only hsCRP and HbA1C maintained a significant negative correlation with %ASM. In non-sarcopenic obese women with PCOS, hsCRP and HOMA were negatively, and 25-hydroxyvitamin D positively correlated with %ASM. These results suggest that in non-sarcopenic obese women with PCOS vitamin D may play a protective role against sarcopenic obesity. Furthermore, sarcopenic obesity may be more associated with inflammation versus insulin resistance in women with PCOS. This is supported by a study demonstrating elevated hsCRP in both obese and non-obese women with PCOS, while only obese women demonstrated insulin resistance [42]. In newly diagnosed untreated diabetics, hsCRP appears to be a reflection of adiposity and is not associated with insulin resistance [43]; thus, the increased visceral adiposity and associated inflammation in women with PCOS may be an independent contributor to sarcopenic obesity in this population.

One of the strengths of this study is the inclusion of > 25 follicles in the definition of PCOS which has been suggested to provide a more accurate definition of the syndrome [19]. In contrast, PCOS was not absolutely ruled out in our control population which is a limitation of this study, as examination for hyperandrogenicity and polycystic ovaries was not completed. Another limitation is the use of correlations between metabolic markers and %ASM; causation cannot be assumed. In addition, in our control group we only determined body composition measures and did not assess metabolic markers or waist circumference; therefore, these measures could not be directly compared to the women with PCO. Although we determined a positive correlation between vitamin D and %ASM in women with PCOS, the interpretation of this result and other correlations is limited given comparison with control correlations cannot be made and results cannot be assumed in this group. Longitudinal studies are required to determine whether changes in metabolic markers over time or with treatment result in increased ASM in women with PCOS. Although our PCOS and control group were recruited from the same population, genetic heterogeneity among the population may have affected our results. A final limitation of the study is that our results are specific to women between the ages of 18 and 35y and therefore not generalizable to ages outside this range.

## Conclusion

In conclusion, we report the prevalence of sarcopenic obesity to be 53% in women with PCOS, which is higher than the prevalence in older women reported from the literature. The correlation of %ASM with hsCRP in both sarcopenic obese and non-sarcopenic obese women with PCOS suggests inflammation may play a primary contributing factor to the high prevalence of sarcopenic obesity in this population. Future studies should focus on whether reduction of inflammatory markers can reduce the prevalence of sarcopenic obesity in women with PCOS.

## Abbreviations

ASM: Appendicular skeletal muscle mass
BFLTM: Bone-free leant tissue mass
BMI: Body mass index
FM: Fat mass
HbA1C: Glycosylated hemoglobin
HOMA: Homeostasis model assessment insulin resistance index
hsCRP: High sensitivity C-reactive protein
PCOS: Polycystic ovary syndrome
VDR: Vitamin D receptor
